# Recent Advances on the Individual Roles and Emerging Synergistic Effects of Plant Growth-Promoting Rhizobacteria and Silicon Nanoparticles in Mitigating Salinity Stress

**DOI:** 10.3390/plants14233632

**Published:** 2025-11-28

**Authors:** Sajida, Hamdy Kashtoh, Tensangmu Lama Tamang, Kwang-Hyun Baek

**Affiliations:** 1 Department of Botany, Hazara University, Mansehra 21300, Pakistan; sajidatoori1@gmail.com; 2 Department of Biotechnology, Yeungnam University, Gyeongsan 38541, Gyeongbuk, Republic of Korea; hamdy_kashtoh@ynu.ac.kr

**Keywords:** abiotic stress, salt tolerance mechanisms, ion homeostasis, gene regulation, sustainable agriculture

## Abstract

Soil salinity is a serious abiotic stressor threatening global agriculture, currently affecting nearly 20% of irrigated land, with projections suggesting that almost 50% of cultivated areas may be impacted by 2050. Plant growth-promoting rhizobacteria (PGPR) and Silicon (Si) have been widely investigated for their individual roles in improving plant tolerance to salinity, yet their combined application—particularly using Si nanoparticles (SiNPs), remains underexplored. This review synthesizes current knowledge on PGPR, SiNPs, and their synergistic effects in mitigating salinity stress, with emphasis on physiological, biochemical, and molecular mechanisms. Special attention is given to Si-mediated regulation of stress-responsive genes (e.g., *RD29B*, *DREB2b*, *RAB18*, *HKT1*, *WRKY TFs*, *CAT*, *POD*) and PGPR-induced gene expression (e.g., *GmST1*, *GmLAX3*, *NHX1*, *NRT2.2*, *GR*), which are directly linked to ion homeostasis, osmolyte accumulation, and antioxidant activation. In addition, crop-specific case studies and emerging molecular insights are highlighted to demonstrate practical applications. Despite these promising findings, significant challenges remain, including the stability of nanoformulations, microbial compatibility, and the lack of field-scale validation under diverse agro-climatic conditions. This review highlights knowledge gaps and briefly outlines future directions for the integrated use of PGPR and SiNPs as sustainable strategies to enhance crop resilience under salinity stress.

## 1. Introduction

The global population is continuously increasing, whereas food crop production is declining due to various climate change, anthropogenic activities, and multiple abiotic stresses such as low temperature, heat, drought, toxic metals, pollution, and salinity [1,2,3]. Among these stressors, soil salinization has emerged as one of the most serious environmental challenges, threatening food security. It affects approximately 7% of global land and nearly one-third of irrigated areas, resulting in severe stress in crops [4]. Salinity and other harmful abiotic factors contribute to the degradation of arable land, rendering it unproductive. Salinity significantly alters plant growth and metabolism, leading to physiological, morphological, and biochemical changes that impair overall plant development [5]. Globally, more than 800 million hectares of cultivable land are already affected by salinity, and the problem continues to expand each year [6,7]. Salinity affects agricultural productivity, threatening both food security and soil health. Currently, about 20% of the world’s irrigated lands are affected by salinity [8,9], and this degradation process is projected to impact nearly 50% of cultivated land in 2050 [10].

Salinity is characterized by the accumulation of soluble salts in the rhizosphere. Nearly 70% of yield reduction in cereal crops such as barley, sesame, sorghum, wheat, and rice is attributed to salt accumulation in agricultural soils, which disrupts plant physiology and metabolism [11,12]. Salinity impairs plant development by causing sodium-induced toxicity and interfering with the absorption of crucial nutrients like calcium (Ca) and potassium (K) [13]. High salinity not only reduces germination and growth but also causes ionic toxicity, osmotic imbalance, and oxidative stress in plants [14,15]. For instance, in faba bean, salinity stress markedly reduced pod production, plant growth [16], photosynthesis, and nutrient absorption, and also delayed flowering [17]. Elevated sodium ion (Na^+^) concentrations disrupted the Na^+^/K^+^ ratio and slowed down cell division, disturbing osmotic balance [1].

Silicon (Si) is the second largest element in the Earth’s crust, making up about 28.8%. It commonly occurs in the form of silicates or metasilicates. Si content in most soils ranges from 14 to 20 mg/L [18]. Certain plant species, such as barley, rice, and wheat, are known to accumulate Si in the form of silicic acid within their tissues, which enhances cell wall strength and imparts structural elasticity [19,20,21]. While crops such as millet, rice, maize, and wheat naturally contain significant amounts of Si, external Si supplementation under stress conditions has been shown to further improve plant growth and grain yield [22,23]. Si plays a vital role in enhancing plants’ resilience to abiotic stresses and improving the yield and quality of grains, vegetables, seeds, and fruits [24]. Si is absorbed by plant roots from the soil and contributes to plant defense by providing protection against insects, pests, and microbial pathogens [25]. Other forms of Si, such as Silicon dioxide (SiO_2_) and Si nanoparticles (SiNPs), ranging from 10 to 100 nm in size, are readily absorbed by plants and can serve as carriers for fertilizers and essential metal nutrients [26,27]. Although the exact mechanism of Si action remains unclear, it is believed to enhance nutrient use efficiency, thereby promoting plant growth, photosynthetic activity, and antioxidant responses [28]. Si is taken up by plants via specific transporter proteins and enhances the absorption of key nutrients, including magnesium (Mg), phosphorus (P), nitrogen (N), and K, ultimately contributing to improved grain development in various crops [28]

Plant Growth-Promoting Rhizobacteria (PGPR) is a collective term used to describe beneficial bacteria that have a symbiotic association with plant roots [29,30]. These microbes enhance plant growth by producing phytohormones, improving nutrient uptake, and combating plant diseases [31]. PGPR are well known for supporting plants under stress by influencing various physiological pathways [31,32,33]. Studies have demonstrated that PGPR inoculation helps reduce the adverse effects of salinity stress [34,35]. Application of PGPR in salt-stressed plants enhances ion uptake, maintains a balanced Na^+^/K^+^ ratio, boosts antioxidant enzyme activity, and increases microbial diversity in the rhizosphere [36,37]. Thus, the introduction of PGPR into saline soils represents an eco-friendly and sustainable strategy to improve crop performance [38].

Recent studies have suggested that Si supplementation together with PGPR inoculation may provide enhanced salinity tolerance through improved ion homeostasis, antioxidant activation, and regulation of stress-responsive genes [39,40]. For instance, Xu et al. found that the combined use of Si and *Enterobacter* sp. FN0603 significantly improved the salinity stress tolerance of wheat plants and greatly enhanced plant growth compared to treatments with either Si or FN0603 alone, demonstrating a synergistic effect on promoting plant growth [41]. A significant abundance of strain FN0603 was observed in the roots of treated plants, and the addition of Si further boosted its colonization in the roots of wheat under salt stress. Furthermore, FN0603, especially when applied alongside Si, altered the bacterial and fungal communities inside the roots rather than those in the rhizosphere. Similarly, Al-Garni et al. reported that the co-application of PGPR and Si resulted in improved resilience to salinity stress in coriander plants compared to using each treatment individually [42]. These crop-specific examples demonstrate that integrated applications can effectively alleviate the deleterious effects of salinity stress, offering a promising method for sustainable agriculture in saline soils. Consequently, further research is required to elucidate their synergistic roles in enhancing salinity tolerance.

Despite extensive research on the individual roles of Si and PGPR in alleviating salinity stress, relatively few studies have explored their combined use. There are limited research studies, often restricted to laboratory or greenhouse studies, which leave a major gap in understanding how these two strategies may act synergistically in real agricultural systems. This review aims to compile existing knowledge on the individual and combined roles of Si and PGPR in enhancing salinity tolerance, highlight recent advances in their integrated application, identify existing knowledge gaps, and outline future research directions. Particular emphasis is placed on their physiological, biochemical, and molecular mechanisms, including gene expression regulation, to highlight their potential as sustainable and eco-friendly strategies for improving crop resilience under saline conditions.

## 2. Role of Si in Salinity Stress Tolerance

Si, not initially recognized as vital for plant function, has garnered increasing attention for its important role in alleviating the negative impacts of environmental stresses like salinity [43,44]. Si fertilization has increasingly attracted attention due to its dual function in mitigating salt stress and enhancing soil health. Si reinforces plant cell walls by promoting silicification, thereby reducing Na^+^ influx and alleviating osmotic stress [45]. Si has been widely recognized for its capacity to boost plant tolerance against salinity stress by modulating both physiological and biochemical processes [46].

Si application in plants has been shown to enhance water retention, strengthen cell wall structure, increase chlorophyll content, and improve photosynthetic efficiency, thereby supporting better growth under saline conditions [47]. Si further enhances the antioxidant defense system by upregulating activities of enzymes such as peroxidase (POD) and catalase (CAT), ultimately reducing malondialdehyde (MAD) accumulation, a key marker of lipid peroxidation and membrane damage [48]. In addition, it influences microbial communities of soil by increasing the presence of beneficial groups such as *Actinobacteria* and nitrogen-fixing bacteria, which in turn promote nutrient cycling and improve crop yield [24,49].

Studies have demonstrated the effectiveness of Si in improving salt tolerance across various crops, including rice, maize, and soybean [50]. Si can be applied in plants through foliar application, growth medium amendment, and soil drenching [51]. Among these methods, foliar application has been found to be the most efficient in attenuating the detrimental effects of salinity stress [52]. Various Si-based materials have been assessed to mitigate salinity stress [53]. Among them, nano-Si has gained attention in agricultural applications due to its extremely small particle size, increased surface area, and superior bioavailability, which improve nutrient uptake [54,55]. Moreover, seed priming with SiNPs has been shown to improve germination performance and physiological function, thereby enhancing crop adaptation to abiotic stress [56].

### 2.1. Si-Mediated Physiological and Biochemical Mechanisms Under Salinity Stress

Si supplementation has emerged as an intriguing approach to reduce the hazardous impacts of salinity on plants. It is widely applied as a component of fertilizers to enhance plant resilience under stress conditions [57]. Moreover, Si enhances photosynthetic efficiency, minimizes transpiration, and improves plant tolerance to salinity stress by regulating oxidative stress responses, cellular homeostasis, and photosynthetic processes [58,59].

One of the key roles of Si in salt-stressed plants is the alleviation of osmotic stress and ionic toxicity. Si improves the capacity of plants to withstand high salinity by reducing Na^+^ uptake while maintaining an optimal Na^+^/K^+^ balance [60]. During active growth, plants absorb Si more rapidly than water, which helps in lowering the concentration of Na^+^ in plant tissues [61]. Furthermore, Si deposition in roots reduces the accumulation of toxic ions, enhances water uptake through aquaporins, and improves root hydraulic conductance. These changes collectively contribute to higher chlorophyll levels, better relative water content (RWC), and improved photosynthetic performance [62]. The contribution of Si to salt stress alleviation has been demonstrated in many plant species [63,64]. Moreover, its strong antioxidant properties enable plants to neutralize excessive reactive species (ROS), thereby preventing cellular damage and minimizing growth inhibition under saline conditions [65].

Laifa et al. investigated the impacts of salinity on sea barley and studied the role of Si in mitigating these effects [66]. When exposed to 150 mM NaCl, the plants showed a significant reduction in growth, pigment content, photosynthetic performance, and increased lipid peroxidation. However, supplementing the growth medium with 0.5 mM Na_2_SiO_3_ effectively alleviated these negative effects by improving growth parameters, increasing chlorophyll content, and reducing oxidant damage. Notably, Si provided specific protection to the photosystem I (PSI) machinery, highlighting its critical role in enhancing salinity tolerance in sea barley [66]. Similarly, a metabolomic analysis of Si effects in tomato plants under salt stress demonstrated that the beneficial effects were linked to the modulation of key primary metabolic pathways, including those participating in the biosynthesis of various amino acids, tricarboxylic acid, and fatty acid [67]. A study on *Zinnia elegans* demonstrated that Si supplementation imparted salt stress tolerance by maintaining osmotic balance, and modulation antioxidant enzyme activity [68]. Overall, Si supplementation has shown potential in mitigating salinity stress across various crops by regulating physiological and biochemical processes, including membrane stability, ionic balance, antioxidant defense, redox homeostasis, and metabolomics adjustments [69].

### 2.2. Effects of SiNPs on Crops Under Salinity Stress

SiNPs possess distinct characteristics such as a nanoscale size and increased surface area that make them effective than conventional bulk Si. For instance, exogenous application of SiNPs to *Elymus sibiricus* under saline conditions led to a notable 96% increase in protein content [70]. In tomato, nano-Si has been shown to enhance seed germination and early growth by regulating the activity of salt tolerance-related genes [71]. Similarly, SiNPs effectively reduced the harmful effects of salinity and promoted the growth of boysenberry plants [72]. SiNPs application in salt-stressed banana plants led to improved chlorophyll content, photosynthetic efficiency, and K accumulation [73]. Badaway et al. observed that exogenously applied SiNPs enhanced water retention and ion homeostasis in rice plants under salt stress, leading to better plant vigor [74]. Similarly, Faizan et al. revealed that supplementing with nano-Si increased concentrations of photosynthetic pigments and alleviated the harmful impacts of salinity on rice plant growth [75]. These results highlight the potential of nano-Si as an efficient and eco-friendly strategy to enhance salt tolerance in various crops.

**Figure 1:**
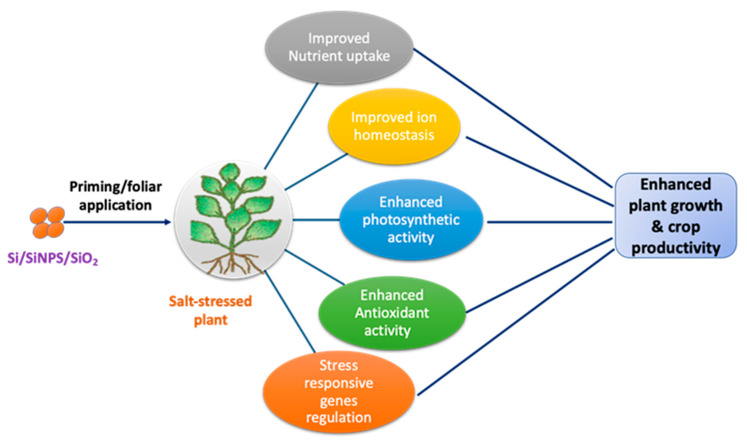
General mechanisms by which SiNPs treatment enhances plant resilience under salinity stress.

Nano-Si application has been linked with boosted antioxidant defense mechanism and osmolyte accumulation. Elevated activities of key antioxidant enzymes like POD, CAT, and SOD, along with increased levels of ascorbic acid and glutathione, were observed in rice seedlings under salinity stress, contributing to improved physiological status [76]. Additionally, nano-Si treatments aided in the regulation of ion homeostasis, particularly Na^+^/Cl^−^ and Na^+^/K^+^ ratios, thereby facilitating better nutrient uptake and growth in saline conditions [77]. At the molecular level, SiO_2_ nanoparticles modulated stress signaling pathways, interacted with ROS, and influenced stress–responsive gene expression [78]. These promising findings emphasize the effectiveness of nano-Si as a promising technique to strengthen salinity tolerance in diverse crop species [73]. An overview of the mechanisms by which SiNPs improve plant resilience under salinity stress was depicted in Figure 1.

Studies have revealed that priming seeds with Si enhances germination rates and reduces the deleterious effects of salinity in wheat [79]. Hasanaklou et al. evaluated the impact of different concentrations of bulk and nano-sized Si materials on seed germination of *Stevia rebaudiana* [80]. Notable enhancements in germination percentage (106%) and germination rate (128.12%) were observed. Improvements were also recorded in growth traits such as root and shoot lengths, as well as seedling dry biomass. It was found that seed priming with nano SiO_2_ at a concentration of 10 ppm was the most effective, whereas the higher doses led to stress symptoms, marked by elevated hydrogen peroxide content, suggesting oxidative damage. This highlights the significance of using optimal dose of Si in seed priming to maximize plant health and growth.

Alsamadany et al. [56] reported that seed treatment with SiNPs successfully alleviated the negative impact of sodium oxide–induced alkaline stress in maize. The treatment led to significant improvements in plant development traits, including improved water-holding capacity, and elevated levels of light-harvesting compounds, readily available proteins and sugars, essential minerals like potassium, and unbound amino acids. Additionally, it reduced the accumulation of proline and Na, resulting in a more favorable Na^+^/K^+^ balance, contributing to enhanced stress resilience. Nevertheless, the impact of seed treatment with SiNPs varies across the plant species and types of abiotic stress involved (Table 1). For example, in *Brassica juncea*, treatment with Si-based nanoparticles enhanced specific developmental traits but did not significantly affect seed yield or oil content [81]. Therefore, while SiNPs, especially when used in seed priming, have demonstrated effectiveness in improving crop performance under numerous abiotic stresses, their dosage and application strategies must be tailored according to the crop species and specific environmental context.

### 2.3. Silicon—Mediated Gene Expression

Induced mutagenesis studies in rice have identified the *Lsi1* gene, an influx transporter of the NIP III aquaporin subfamily. *Lsi1* contains a Gly-Ser-Gly-Arg (GSGR) motif within its membrane filter, which selectively facilitates Si uptake. The amino acid composition between NPA (Asn-Pro-Ala) domains plays a vital role in determining Si accumulation in plants, while the 108-amino-acid GSGR filter modulates monosilicic acid permeability. *Lsi2*, a Si efflux transporter, functions as an H^+^ antiporter with 9–12 transmembrane domains. In rice, monosilicic acid enters the distal exodermis via *Lsi1* and is transported toward the aerenchyma by *Lsi2* at the proximal side [51,87]. The upward movement of Si to aerial tissues occurs via transpiration flow, while *Lsi6* mediates its redistribution from the xylem to adjacent tissues (Table 2).

In maize, Si deposition is controlled by ZmLsi1, which is responsible for root uptake, and ZmLsi2, which facilitates xylem loading. In contrast to *Lsi2*, in barley, HvLsi2 does not exhibit polar localization and restricts Si movement within the endodermis. Additionally, *Lsi3*, an efflux transporter homologous to *Lsi2*, has been identified in rice [51]. Localization of *Lsi2*, *Lsi3*, and *Lsi6* at stem nodes highlights their function in intravascular Si transport.

Comparative genomic analyses of soybean, rice, and *Arabidopsis* using phylogeny, transcriptomics, protein modeling, and gene organization have revealed key intrinsic proteins [87]. In soybean, *GmNIP2-1* and *GmNIP2-2* were identified and cloned as functional Si transporters, indicating conservation of Si transporter genes across species. Interestingly, when orthosilicic concentrations exceed 2 mM, polymerization into SiO_2_ can occur, causing cellular toxicity [88]. Si polymerization associated with modified structures may play a role in pathogen defense in grasses, suggesting that genetic regulation of Si uptake and selective accumulation is crucial for plant adaptation and classification [89].

In addition to transporter-mediated uptake, Si regulates gene expression under salt stress. In maize, Si application reduces Na^+^ accumulation in roots while enhancing its uptake in leaves by upregulating SOS genes, which promote Na^+^ exclusion and facilitate its loading into the xylem [90]. Simultaneously, Si down-regulates *ZmHKT1*, thereby reducing Na^+^ unloading and increasing sequestration into vacuoles, ultimately lowering Na^+^ toxicity in chloroplasts. These changes shift the physiological behavior of maize toward that of halophytes, thereby enhancing tolerance to salinity stress. In rice, overexpression of *OsSOS1* leads to improved chlorophyll content, ion homeostasis, seed germination, seedling survival, and growth under saline conditions, while Si supplementation further amplifies these effects by upregulating *Lsi1*. [51]. Moreover, metabolite profiling revealed the accumulation of osmolytes and amino acids in response to Si, indicating its broader role in coordinating ion transport, gene regulation, and metabolic adjustments essential for salinity tolerance [41].

## 3. Role of PGPR in Salinity Stress Mitigation

### 3.1. General Mechanisms of PGPR

**Figure 2:**
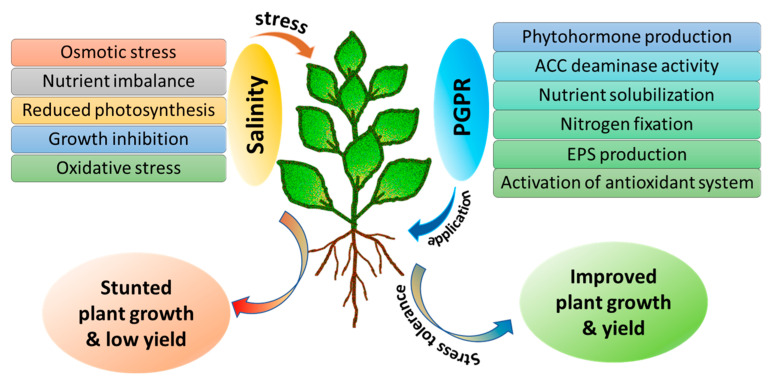
Schematic representation of salinity effects and the role of PGPR in alleviating salinity stress.

PGPR are beneficial soil microbes that inhabit the rhizosphere and enhance plant growth through a wide range of biochemical and physiological mechanisms, acting either directly or indirectly. Salt stress negatively impacts plants by disrupting nutrient balance, lowering photosynthetic efficiency, and triggering excessive accumulation of ROS, which collectively hinder crop growth and reduce yield [91]. According to Pacifico et al. [92], microorganisms support plant development via multiple strategies, such as improving nutrient uptake and assimilation, increasing photosynthetic efficiency, osmolyte accumulation, improving leaf water relations, and strengthening the antioxidant defense system. As a result, they help reduce transpiration, increase stomatal resistance, and regulate key genes connected with the detoxification of ROS. For example, endophytic bacteria such as *Pseudomonas phytofirmans*, *P. migulae*, and *P. fluorescens* produce 1-aminocyclopropane-1-carboxylate (ACC) deaminase enzyme, which prevents the buildup of ethylene, thereby reducing the growth inhibition typically caused by ethylene under salt stress [92]. Backer et al. [93] reported that PGPR modulate the biosynthesis and signaling of several phytohormones, including auxins, ethylene, brassinosteroids, gibberellins, abscisic acid (ABA), cytokinins, and collectively, these hormones govern key aspects of growth, differentiation, and environmental adaptability in plants. Habib et al. [94] reported that ACC deaminase production by PGPR enhances salt tolerance in okra by increasing the activities of antioxidant enzymes such as ascorbate peroxidase and CAT, and by upregulating genes involved in the ROS detoxification pathway. Similarly, Chen et al. [95] demonstrated that *Bacillus amyloliquefaciens* improves saline tolerance in maize by increasing chlorophyll content, total soluble sugars, POD and CAT activity, as well as glutathione content, while simultaneously decreasing Na^+^ accumulation. Recent findings by Win et al. [96] demonstrated that the combined application of *Pseudomonas* spp. and *Bradyrhizobium* spp. Significantly alleviated salt stress in soybean, underlining the potential of rhizobia as PGPR to enhance salt-stress tolerance in this crop. These findings confirm that PGPR play a critical and multifaceted role in enhancing plant resilience to salinity stress through hormonal modulation, antioxidant activity, and improved physiological responses. An illustration of the effects of salinity and the role of PGPR in mitigating salinity stress is depicted in Figure 2.

### 3.2. Physiological Mechanisms of PGPR

PGPR plays a crucial role in enhancing plant growth and development, especially under salt stress conditions. They alleviate the harmful effects of salt by improving root growth, nutrient uptake, and photosynthetic efficiency. For instance, seed inoculation with PGPR has been shown to reduce ethylene accumulation [97], enhance nutrient absorption, and boost photosynthesis [98]. In leguminous crops and mungbean, PGPR application significantly improves seedling growth, final yield, and soil fertility [99,100,101].

Additionally, PGPR helps regulate phytohormone activity, root development, and nutrient transport, leading to better lateral root branching and root hair formation [102]. In crops like pigeon pea and soybean, PGPR improve water uptake, photosynthetic rate, and yield under saline conditions [103].

Several studies [104,105] have confirmed that seed treatment with *Bradyrhizobium japonicum* and *Pseudomonas* enhances shoot biomass, root nodulation, and nutrient absorption. PGPR also strengthens the plant’s stress response by boosting antioxidant enzyme activity and modulating hormone signaling [106].

Beyond growth promotion, PGPR also plays a vital role in sustainable agriculture by reducing reliance on chemical pesticides and fertilizers [107,108]. They have been shown to alleviate salinity stress via diverse physiological and molecular mechanisms [109]. For example, the use of *Enterobacter* sp. P23 and *Pseudomonas* sp. OFT5, both of which possess ACC deaminase activity, helped reduce salinity stress in rice and tomato by lowering production of stress-related ethylene [110,111]. Similarly, *Azospirillum lipoferum* FK1 increased the salt stress resilience of chickpea by adjusting osmolytes accumulation, activating antioxidant enzymes, and regulating stress-responsive genes [112]. In another case, *Enterobacter* sp. SA187 improved salt tolerance in *Arabidopsis thaliana* by reprogramming its sulfur metabolism [113]. Additionally, *Bacillus xanthoxyli* facilitated the removal of excess Na^+^ from the shoots of tall fescue and promoted K^+^ uptake in roots, thereby maintaining ion homeostasis under saline conditions [114].

### 3.3. Mechanisms of Salt-Tolerant PGPR (ST-PGPR) in Enhancing Plant Salt Tolerance

Salt-tolerant PGPR (ST-PGPR) can thrive in saline environments and support crop growth either directly or indirectly under salt stress [115]. PGPR are widely innoculated to improve crop performance, with their modes [116] of action primarily involve: (1) production of phytohormones such as ABA, cytokinins, auxins [117], and indole-3-acetic acid (IAA) which promotes root elongation and boosts nutrient absorption; (2) solubilization of phosphorous and other nutrients, thereby improving soil fertility; and (3) induction of antioxidant enzymes, including SOD, that alleviate oxidative stress caused by excessive ROS and PGPR also produce ACC deaminase, which converts ACC into alpha-ketobutyrate and ammonia, thereby reducing ethylene accumulation in plants. As a result, PGPR can help mitigate salt-induced growth inhibition by lowering endogenous ethylene levels under stress [116]. ST-PGPR-derived gibberellins enhance root hair development, improving nutrient and water uptake [109] and thereby boosting plant salt tolerance [118]. Moreover, ST-PGPR helps alleviate oxidative damage under salinity stress by producing antioxidant enzymes such as SOD [119]. Despite these beneficial effects, their practical application in agriculture remains challenging due to microbial instability, susceptibility to environmental fluctuations, and the inactivation of single-strain formulations. These limitations often result in inconsistent field performance, particularly under diverse agro-climatic conditions. Therefore, future research should emphasize the development of multi-strain consortia, nano-formulation approaches, and genetic engineering tools to enhance the stability, efficiency, and adaptability of ST-PGPR for sustainable crop production under saline environments.

### 3.4. PGPR- Mediated Gene Expression

PGPR are known to modulate plant gene expression, leading to the enhanced synthesis of stress-protective compounds such as ROS-detoxifying enzymes and osmolytes. Functional genes within PGPR, including those involved in IAA secretion (*iaaM*), phenazine biosynthesis (*phzCEF*), nitrogen fixation (*nifU*), siderophore production (*sbnA*), and spermidine synthesis (*speB*), have been directly associated with improved plant growth and stress tolerance (Table 3) [120]. Furthermore, Khan et al. [121] demonstrated that the expression of the salt tolerance gene (*GmST1*) and the IAA-mediating gene (*GmLAX3*) was significantly upregulated in soybean following treatment with halotolerant rhizobacterial strains under salinity stress in *Glycine max*.

Kim et al. [123] demonstrated that *Enterobacter* sp. inoculation in *Arabidopsis* enhanced salt tolerance by triggering salt stress-responsive signaling pathways. Specifically, *DREB2b*, *RD29A*, and *RAB18* were upregulated by EJ01, activating both ABA-dependent and ABA-independent pathways, even in the absence of salinity. Moreover, the expression of *RD29B*, a well-known stress marker gene, was highly induced under saline conditions. Similarly, *P5CS1*, a proline biosynthesis gene, was also elevated, contributing to osmotic adjustment. In another study, Chen et al. [95] found that inoculation with *B. amyloliquefaciens* SQR9 upregulated Na^+^/H^+^ antiporter and N^+^-PPase genes, facilitating vacuolar Na^+^ sequestration, while the expression of *HKT1*, a high-affinity K^+^ transporter, was suppressed in maize, thereby limiting Na^+^ translocation from shoot to root.

The expression of antioxidant enzymes POD and CAT encoding genes was found to be highest in *P. simiae* and sodium nitroprusside-treated soybean plants [124]. Similarly, genes related to auxin, cytokinin, and gibberellic acid signaling pathways were significantly upregulated in *Paenibacillus polymyxa* YCO136-treated tobacco plants compared with uninoculated controls. This was accompanied by enhanced expression of transcription factors [125] such as *WRKY* and *MYBB*, which are well known for their critical roles in gene regulation, growth, development, and plant stress responses [126,127]. Consistent with these observations, several studies have also reported alterations in the expression of *WRKY TF* genes under salinity conditions, particularly in root transcriptomes bacterized with *Azospirillum brasilense*. In these plants, various hormone-related genes (e.g., ACC oxidase, ethylene insensitive genes, auxin efflux carriers, auxin-responsive genes, and cytokinin biosynthesis-related enzymes) were significantly upregulated, along with TF families such as *WRKY*, *MYB*, *AP2/ERF*, and *GRAS* [128].

Furthermore, inoculation with *Dietzia natronolimnaea* under salinity stress resulted in elevated expression of *MYB* and *WRKY TFs*, while nine genes involved in the salicylic acid pathway and six genes that encode phenylalanine ammonia-lyase, a key enzyme in the phenylpropanoid metabolism, were also strongly upregulated [129]. Similarly, inoculation of sugarcane plantlets with *Burkholderia anthina MYSTP113* induced the expression of genes associated with phenylpropanoid and amino acid biosynthesis [130]. In barley, *Arthrobacter nitroguajacolicus* inoculated roots under salt stress showed upregulation of Fe uptake and phosphatase encoding genes, together with enhanced expression of several transporters related to ion, sugar, and amino acid transport [131]. These findings collectively highlight that PGPR inoculation strongly modulated plant transcriptomic responses by activating stress-related signaling pathways, transcription factors, and metabolic genes, thereby enhancing tolerance to salinity stress. Under salt stress, *B. japonicum* suppresses key genes in shoots, such as *DAHAR*, *MSD1*, *MYC2*, *RD22*, and roots like *ADC2*, *ANACO55*, *DHAR*, *GTR1*, *RD20*, *RD29B*, *VSP1*, and *V2p2* [132]. Similarly, *CNBG-PGPR-1* lowered salt stress-induced ROS by stimulating genes involved in glutathione and peroxidase pathways, closely linked to methionine metabolism [133]. For evaluating barley growth under salt stress, two PGPR strains, *Pseudomonas putida* KT2440 and *Pseudomonas fluorescens SBW25*, were selected. qRT-PCR analysis revealed that *P. putida* KT2440 strongly upregulated key Jasmonic acid pathways genes (*FAD3*, *LOX1*, *AOS*, *AOC*) under salt stress. The *MAPKK* gene showed pronounced induction under high salt conditions. Additionally, stress-responsive genes such as *NHX1*, (*P. putida* KT2440), *NR2.2* (*P. fluorescens* SBW25), *CAT2* (*P. fluorescens*), and *GR* (*P. putida*) were also activated under various stress conditions (Table 4) [134].

## 4. Synergistic Effects of PGPR and SiNPs on Salinity Tolerance

**Figure 3:**
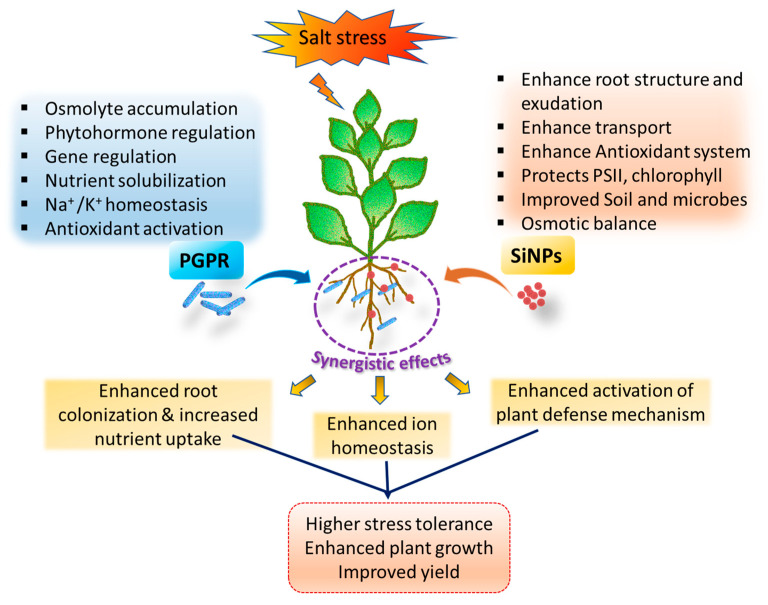
Diagrammatic representation of synergistic effects of SiNPs and PGPR in salt stress tolerance.

The combined supplementation of PGPR and SiNPs consistently produces synergistic effects, resulting in enhanced plant growth and yield compared to the application of either approach alone [41]. This beneficial interaction is attributed to several mechanisms; SiNPs support better root colonization by PGPR by creating a favorable microenvironment and promoting bacterial attachment, while PGPR, in turn, assist in the uptake and translocation of SiNPs within plant tissues [137,138]. This combined strategy has been shown to significantly improve plant resilience against various abiotic stresses, particularly salinity, as well as drought and heavy metal toxicity [139,140,141]. Although studies combining PGPR and SiNPs are limited, the positive effects observed in independent applications suggest strong potential for co-application as a sustainable strategy to enhance plant tolerance to salinity stress [142]. An illustration of the synergistic effects of SiNPs and PGPR on plant tolerance to salt stress is depicted in Figure 3.

The integrated application of PGPR and SiNPs provides an effective strategy for enhancing salinity stress tolerance, especially in crop plants. For example, co-inoculation of Si and *Pseudomonas psychrotolerans* enhanced Si uptake fourfold and lowered Na^+^ accumulation by 42.3% in wheat plants compared to untreated ones. This co-application also promoted overall plant growth more effectively than either treatment alone, highlighting the synergistic effect in mitigating salt stress [143]. Similarly, another study described that under salinity stress, the combined application of SiNPs and biofertilizers containing *Flavobacterium* and *Pseudomonas* spp. significantly improved the physiological and biochemical traits of wheat, including soluble sugars and proteins, leaf water potential, and activities of antioxidant enzymes. This combined treatment was the most effective, enhancing yield and reducing oxidative stress markers, thereby indicating that co-application can alleviate the harmful impacts of salinity in wheat [82]. The application of nano-Si and PGPRs (*Pseudomonas koreensis* and *Bacillus coagulans*) in the salt-sensitive rice variety Giza 177 significantly outperformed the untreated salt-tolerant cultivar Giza 179, in key yield components such as grain yield, number of grains per panicle, and nutrient uptake. Both PGPRs and nano-Si exhibited synergistic and stimulatory effects that alleviated the detrimental impacts of salinity, leading to improved plant growth and grain yield [144]. Field trials showed that co-inoculation of *Azotobacter chroococcum*, *P. koreensis*, and SiNPs remarkably enhanced soil physiochemical properties, particularly by lowering exchangeable sodium, and more effectively ameliorated the harmful effects of saline irrigation on barley than individual applications of PGPR or SiNPs [145]. These findings highlight that the combined application of Si with PGPR could be a promising strategy for irrigating crops with low-quality or saline water in salt-affected soils, particularly in regions with limited freshwater availability.

Besides crops, the application of Si and PGPR has been shown to significantly enhance salinity tolerance in other plants by improving multiple physiological and biochemical traits. For example, in Pak choi, the combined treatments with salt-tolerant PGPR and Si enhanced osmolytes accumulation (proline and soluble proteins), antioxidant enzymes activities (CAT, POD, SOD), and regulated phytohormones (salicylic acid, Jasmonic acid, and ABA), which collectively reinforced stress tolerance [146]. Similarly, in melon seedlings, the combined application of Si and PGPR consortium (*Agrobacterium* sp., *Pantoea* sp., and *Priestia* sp.) significantly boosted plant growth indices, improved rhizosphere nutrient levels, and enhanced enzyme activities compared to individual treatments. This indicates that integrating Si with PGPR offers a promising and sustainable approach to mitigate salinity stress through complementary mechanisms [147]. Likewise, under the combined stress from saline water irrigation and high soil salinity, co-application of PGPR and Si enhanced antioxidant activity, decreased Na^+^ uptake and oxidative stress, subsequently leading to enhanced root growth and sugar yield in sugar beets [148]. Adhikari et al. evaluated the effects of *P. koreensis*, Si, and phosphorus on soybean under salt stress. Compared to the sole application, their combined application significantly improved nutrient uptake, lowered Na⁺ influx, increased K⁺ uptake, and upregulated salt-resistance genes (*GmSALT3*, *GmST1*, *GmAKT2*) [149]. Several studies have exhibited the synergistic effects of Si and PGPR in enhancing salinity stress tolerance across various plant species (Table 5).

A two-year field study on mung bean grown under saline irrigation showed that the co-application of Si and PGPR enhanced salinity tolerance compared to control or individual treatments by regulating osmolytes, reducing leaf lignification, improving minerals uptake (K^+^, Ca^2+,^ and Si), and lowering tissue Na^+^ content [150]. *Bacillus drentensis* and Si treatment remarkably alleviated salinity-induced oxidative stress in mung bean leaves compared to sole applications [39]. In addition, physiological traits, growth, and yield were significantly improved under salinity stress during a two-year field trial [158]. ACC deaminase-producing PGPR combined with Si significantly enhanced nodule and leaf number as well as grain yield in mung bean, while reducing Na⁺ accumulation, highlighting its effectiveness in mitigating salinity stress [159]. Kumar et al. used response surface methodology to optimize PGPR consortia and Si fertilization for *Phaseolus vulgaris* under saline stress. A central composite design assessed the effects of varying PGPR and Si doses on growth, yield, and biochemical traits. Optimized doses (5.52 × 10^7^ cfu/g PGPR and 10.90 g/kg Si) maximized plant height, pod length, and yield, chlorophyll content, and antioxidant enzyme activities, emphasizing the significance of dose optimization for effective co-inoculation of PGPR and Si in saline soils [160].

There are promising positive outcomes reported regarding the combined use of PGPR and Si in alleviating salinity stress in plants. However, these findings are still at a preliminary stage. Further research, particularly field-based studies, is needed to check the actual performance under real environments. In addition, toxicity assessments on the soil ecosystem and other beneficial plant-associated microbes are still lacking. Most studies have used bulk Si, rather than SiNPs, in combination with PGPR. Thus, the precise roles of SiNPs compared with bulk Si in producing synergistic effects are still poorly understood. Moreover, a better understanding of the optimal doses of PGPR and SiNPs under actual saline conditions is essential to ensure soil health and crop productivity.

## 5. Future Perspectives

The co-inoculation of PGPR and SiNPs presents a promising approach for sustainable agriculture, especially in enhancing plant tolerance to abiotic stresses like salinity. Although considerable advancements have been made in studying their synergistic effects, further research is required to fully understand and harness their combined potential. Physiological and biochemical studies have demonstrated that both Si and PGPR improve antioxidant activities, chlorophyll content, osmolytes accumulation, and ion homeostasis under salinity stress [95]. However, the mechanism behind how SiNPs and PGPR interact with plants remains poorly understood. Therefore, further studies are needed to understand how these responses are coordinated at the molecular level. Future research should employ advanced omics tools (transcriptomics, proteomics, metabolomics, ionomics) to elucidate how SiNPs influence PGPR colonization and decipher crosstalk between microbial signaling and Si-mediated pathways [95].

Although SiNPs have shown promising effects on plant growth and stress tolerance, their long-term stability, environmental safety, and microbial interactions remain insufficiently studied [72]. The development of nano-bioformulation systems integrating PGPR with SiNPs using encapsulated carriers that allow gradual release and balance nutrient supply, along with microbial viability, could open new avenues for safe and effective agricultural applications. However, these nano-bioformulations require careful evaluation through toxicity risk assessments and standardized safety guidelines to ensure environmental and ecological safety. Establishing standardized nano-bioformulation protocols and quality control measures will be essential for ensuring farmer acceptance and facilitating wider application. In addition, exploring green-synthesis approaches using plant extracts to synthesize SiNPs could further minimize potential toxicity. Additionally, gene-editing techniques such as CRISPR-Cas9 could be utilized to validate candidate genes and develop crop varieties with enhanced Si uptake and better PGPR compatibility [121].

Another critical gap is that most existing studies on co-inoculation of PGPR and SiNPs have been confined to laboratory or greenhouse conditions. Therefore, future research should focus on conducting long-term field trials to evaluate the performance, durability, and biosafety of combined PGPR–SiNP applications across diverse soil types, climates, and management practices. Collecting data on yield improvement, nutrient-use efficiency, and changes in the soil microbiome will be vital for determining the practicality of large-scale application. Furthermore, adopting a systems biology approach that integrates molecular, biochemical, and agronomic data could accelerate progress. Integrating these approaches with precision agriculture and big data analytics could help create predictive models for crop performance and guide the development of PGPR-Si technologies for climate-smart agriculture [120].

## 6. Conclusions

In conclusion, salinity stress continues to pose a critical threat to global food security, affecting plant growth and productivity. Both PGPR and SiNPs have shown strong potential in enhancing salinity tolerance through modulation of phytohormones, ion regulation, antioxidant defense, and molecular signaling pathways. Recent studies have demonstrated that PGPR upregulates key stress-related genes such as *GmST1*, *GmLAX3*, *NHX1*, and *NR22*, while Si and SiNPs regulate important genes, including *RD29B*, *DREB2b*, *RAB18*, *HKT1*, *WRKY* transcription factors, *CAT*, and *POD* under salt stress conditions. These molecular interactions indicate that the combined use of PGPR and Si reinforces plant defense systems by coordinating osmotic balance, antioxidant activity, and ion transport. However, most of these findings are still confined to greenhouse or laboratory settings, emphasizing the need for field validation. Future research should prioritize large field-scale trials and employ advanced omics approaches to integrate molecular and physiological data, while evaluating biosafety and nano-bioformulation efficiency to fully exploit the synergistic potential of PGPR and Si/SiNPs in mitigating salinity stress. Addressing these challenges could establish bio-nano synergistic approaches as a key strategy for advanced sustainable agriculture systems.

## Figures and Tables

**Table 1:** Reported applications of silicon nanoparticles and their effects on different crops under salinity stress.

Application Type	Crop	Si Form	Concentration	Observed Effect	Ref.
Exogenous application	Rice	SiNPs	Not specified	Enhanced water retention, improved ion homeostasis, and contributed to improved plant vigor under salinity stress	[74]
Seed Priming	Stevia	SiO_2_NPs	Moderate	Increase germination, increase root/shoot dry weight	[80]
Hydroponic Addition	Sea barley	Na_2_SiO_3_	0.5 mM	Increase chlorophyll, decrease oxidative stress	[66]
Seed Priming	*B. juncea*	SiNPs	Optimal	improving plant stress tolerance	[81]
Foliar Spray	Wheat	SiNPs	60 mg/L	Mitigating the salinity stress by enhancing antioxidant activity, improved physiological parameters by stomatal conductance, electrical conductivity, electrolytic leakage, and proline	[82]
Mixed in Soil	Soybean	SiO_2_NPs	1 g/kg of dry soil	Promoted root development, altered root exudation patterns, stimulated the recruitment and colonization of beneficial microbes, and enriched the rhizosphere microbiome	[83]
Seed Priming	chia	SiNPs	200 mg/L	Alleviated salt stress by enhancing antioxidant enzyme activity, maintaining ionic equilibrium, and improving seed yield and oil content	[7]
Mixed in Soil	Tomato	SiO_2_NPs	50 ppm	enhancing antioxidant enzyme activity, Improved proline metabolism, increased osmoregulatory substances, and decreased H_2_O_2_ levels.	[84]
Mixed in Soil	Wheat	SiNPs	10%(optimal)	improved chlorophyll content, proline accumulation, soluble sugars, nutrient content, and water retention, and reduced electrolyte leakage	[85]
Hydroponic Addition	Tomato	SiNPs	200 mg /L	Improved growth, photosynthesis, and chlorophyll content, increased antioxidant enzymatic activity, and reduced H_2_O_2_ levels. Increased K^+^ and Si content and decreased Na^+^ absorption.	[17]
Exogenous application	Rice	SiNPs	200 mM	Alleviated salt stress by enhancing antioxidant enzyme activity, maintaining ionic equilibrium.	[47]
Exogenous application	Wheat	SiO_2_-ZnO NPs	-	Alliviated salt stress, improved growth, yield, and nutrient content.	[86]

**Table 2:** Silicon transporter genes and their functions.

Gene	Plant Source	Function/Role	Localization
*Lsi1*	Rice, Maize	Influx transporter; uptake of Si into root cells	Distal exodermis
*Lsi2*	Rice, Barley	Efflux transporter; exports Si toward xylem	Proximal exodermis
*Lsi3*	Rice	Efflux transporter; assists Si movement within stem nodes	Stem nodes
*Lsi6*	Rice, Wheat	Distributes Si from xylem to aerial tissues	Leaf nodes, shoots
*GmNIP2-1*, *GmNIP2-2*	Soybean	Aquaporin family; function as Si transporters	Root tissues

**Table 3:** PGPR functional gene contributing to plant growth and stress tolerance.

Gene	Function/Role	Plant System/PGPR	Ref.
*iaaM*	IAA secretion	PGPR strains	[120]
*nifU*	Nitrogen fixation	PGPR strains	[120]
*phzCEF*	Phenazine biosynthesis	PGPR strains	[120]
*sbnA*	Siderophore production	PGPR strains	[120]
*speB*	Spermidine biosynthesis salt tolerance gene	PGPR strains	[120]
*GmST1*	Salt tolerance gene	Soybean	[121]
*GmLAX3*	Auxin transport gene	Soybean	[121]
*htpX*	Heat resistant	PGPR strains	[122]
*otsA*	Osmoprotection	PGPR strains	[122]
*katE*	Antioxidant gene	PGPR strains	[122]
*uvrA*	UV radiation resistant	PGPR strains	[122]

**Table 4:** Stress-responsive genes regulated by PGPR under salinity stress.

Gene	Function/Role	Plant/PGPR Strain	Ref.
*P5CS1*	Proline biosynthesis	*Arabidopsis*	[123]
*RD29A/RD29B*	Dehydration responsive genes (ABA dependent/independent)	*Arabidopsis*	[123]
*DREB2b*	Dehydration-responsive element binding TF	*Arabidopsis*	[123]
*RAB18*	ABA-responsive gene	*Arabidopsis*	[123]
*HKT1*	High affinity K^+^ transporter (Na^+^ exclusion)	Rice, Wheat	[135]
*WRKY TFs*	Transcription factors regulating stress tolerance	Multiple crops	[136]
*DAHAR*, *MSD1*, *MYC2*, *RD22*	Salt stress-responsive genes (shoots)	*Arabidopsis* under *B. japonicum*	[132]
*ADc2*, *ANACO55*, *DHAR*, *GTR1*, *RD20*, *RD29B*, *VSP1*, *V2p2*	Salt stress-responsive genes (roots)	*Arabidopsis* under *B. japonicum*	[132]
Glutathione and Peroxidase pathways genes	ROS detoxification, linked to methionine metabolism	*Arabidopsis* with CNBG-PGPR-1	[133]
*FAD3*, *LOX1*, *AOS*, *AOC*	Jasmonic acid pathway genes	Barley with *P. putida* KT2440	[134]
*MAPKK*	MAPK signaling under salt stress	Barley with *P. putida* KT2440	[134]
*NHX1*	Na^+^/H^+^ antiporter	Barley with *P. putida* KT2440	[134]
*NRT2.2*	Nitrate transporter	Barley with *P. fluorescens* SBW25	[134]
*CAT2*	CAT (ROS detoxification)	Barley with *P. fluorescens*	[134]
*GR*	Glutathione reductase	Barley with *P. putida*	[134]

**Table 5:** Synergistic effects of co-inoculation of various PGPR spp. and SiNPs across various crops.

S.N.	PGPR spps.	Si Source	Crops	Co-Inoculation Effects	Ref.
1	*B. drentensis*	K_2_SiO_3_	Mung bean	Enhanced K^+^, Si, and Ca^2+^ concentrations in shoots, lowered Na^+^ content compared to the control, and higher pod yield.	[150]
2.	*P. pseudoalcaligenes*	K_2_SiO_3_	Coriander	Enhanced photosynthetic pigment levels, higher RWC, increased POD activity, and an improved root system contributed to enhanced plant growth.	[42]
3.	*A. lipoferum* and *Bacillus circulance*	SiO_2_	Maize	Significant improvements in soil health and plant growth accompanied by enhanced nutrient uptake, yield-related traits, and maize productivity.	[151]
4.	*Enterobacter* sp.	Na_2_SiO_3_	Wheat seeds	Increased stress tolerance indices, resulting in a drastic improvement in plant growth compared to the individual application of Si or bacteria.	[41]
5.	*Bacillus thuringiensis*	K_2_SiO_3_	Lettuce	Higher head weight, yield, and antioxidant enzyme activities (CAT, SOD, POD, and polyphenol oxidase) with increased proline accumulation in lettuce leaves.	[152]
6.	*Flavobacterium* and *Pseudomonas*, along with arbuscular mycorrhizal fungi	SiO_2_	Wheat	Improved Si, P, and K^+^ levels and lowered Na^+^ uptake, thereby increasing grain yield.	[153]
7.	*Advenella incenata* and *Ensifer meliloti*	K_2_SiO_3_	Alfalfa	Enhanced both morphological and physiological traits of alfalfa plants, lowered Na^+^, and increased K^+^ content.	[154]
8.	*Rhizobium leguminosarum* and *Bacillus circulans*	K_2_SiO_3_	Faba bean	Lowered exchangeable sodium percentage and promoted urease and dehydrogenase activities, with values similar to the control (fresh water), contributing to soil quality restoration and ultimately improving plant growth.	[155]
9.	*Bacillus cereus-Amazcala*	SiO_2_	Chilli pepper	SiO_2_-NPs enhanced PGPB’s phosphate solubilization capacity and GA_7_ production. While upregulating CAT and SOD activities, indicating that SiO_2_-NPs function as a eustressor.	[156]
10.	*Bacillus subtilis*	Nano-SiO_2_	Wheat	Enhanced nutrient content and wheat growth, mitigating the detrimental effects of salinity stress.	[157]
